# Multivariate Profiling of Metabolites and Volatile Organic Compounds in *Citrus depressa* Hayata Fruits from Kagoshima, Okinawa, and Taiwan

**DOI:** 10.3390/foods12152951

**Published:** 2023-08-04

**Authors:** Yonathan Asikin, Yoshio Tamura, Yusuke Aono, Miyako Kusano, Hiroshi Shiba, Masashi Yamamoto, Fumimasa Mitsube, Shu-Yen Lin, Kensaku Takara, Koji Wada

**Affiliations:** 1 Department of Bioscience and Biotechnology, Faculty of Agriculture, University of the Ryukyus, Nishihara 903-0213, Japan; 2 United Graduate School of Agricultural Sciences, Kagoshima University, Kagoshima 890-0065, Japan; 3 Feed and Livestock Production Division, Zennoh, Tokyo 100-6832, Japan; 4 Degree Programs in Life and Earth Sciences, University of Tsukuba, Tsukuba 305-8572, Japan; 5 Faculty of Life and Environmental Sciences, University of Tsukuba, Tsukuba 305-8572, Japan; 6 Tsukuba-Plant Innovation Research Center, University of Tsukuba, Tsukuba 305-8572, Japan; 7 RIKEN Center for Sustainable Resource Science, Yokohama 230-0045, Japan; 8 Department of Agricultural Sciences and Natural Resources, Faculty of Agriculture, Kagoshima University, Kagoshima 890-0065, Japan; 9 Okinawa Prefectural Agricultural Research Center Nago Branch, Nago 905-0012, Japan; 10 Department of Horticulture and Landscape Architecture, National Taiwan University, Taipei 10617, Taiwan

**Keywords:** *Citrus depressa*, Shiikuwasha, metabolites, volatile organic compounds, multivariate analysis

## Abstract

*Citrus depressa* Hayata is a small-fruit citrus species; it is indigenous to Kagoshima, Okinawa, and Taiwan. The metabolites and volatile organic compounds (VOCs) that affect the flavor of its fruits have not been investigated based on geographical origin. In the present study, we investigated the metabolite and VOC profiles of 18 *C. depressa* cultivation lines from these regions. Multivariate analysis revealed differences in the metabolites of *C. depressa* based on its cultivation origins; variations in sugar, sugar alcohol, and amino acid contents were also observed. Fruits from Kagoshima and Okinawa had higher galactinol, trehalose, xylose, glucose, and sucrose intensities than fruits from Taiwan (log_2_-fold change; 2.65–3.44, 1.68–2.13, 1.37–2.01, 1.33–1.57, and 1.07–1.43, respectively), whereas the Taiwanese lines contained higher leucine, isoleucine, serine, and alanine. In contrast to the Taiwanese Nantou line, other cultivation lines had comparable total VOC contents, and the VOCs of all lines were dominated by limonene, *γ*-terpinene, and *p*-cymene. Accordingly, the highest VOC intensities were recorded in the Nantou line, which was followed by Shikunin sweet (Kagoshima) and Taoyuan (Taiwan) (log_10_ normalize concentration; 5.11, 3.08, and 3.01, respectively). Moreover, multivariate analysis plots elucidated the difference in the VOCs of Ishikunibu (Okinawa), Shikunin sweet, and Taoyuan and between those of most Kagoshima and Okinawa cultivation lines. These results suggest that both the cultivation line and origin influence the metabolites and VOCs of *C. depressa*, thus possibly affecting its flavor quality; the data provide a valuable insight for utilizing *C. depressa* of different cultivation lines and origins to produce foods and beverages.

## 1. Introduction

*Citrus depressa* Hayata is a citrus fruit cultivar indigenous to the Ryukyu Archipelago of Japan, specifically the Amami Islands of Kagoshima Prefecture and Okinawa Prefecture, and Taiwan. It is a small variety of citrus fruits and is known by different names, such as Hirami lemon, flat lemon, and thin-skinned flat lemon; locally, it is known as Shikunin and Shikuribu (Kagoshima), Shiikuwasha (Kagoshima and Okinawa), and Biǎn shí níngméng (Taiwan). *C. depressa* is known for its tart and slightly sweet taste and distinct aroma [1,2]. It is often used in Japanese cuisine, particularly in drinks and deserts, and it is also popular in Taiwan [2,3]. The fruit is rich in phytochemicals, such as phenolics, flavanones, and polymethoxylated flavones, with potentially health-enhancing properties [1]. Moreover, the essential oil derived from Okinawan Shiikuwasha, which contains limonene and *γ*-terpinene, ameliorates stress and exerts anti-inflammatory effects [4].

Metabolite profiling including primary metabolites of fruits, particularly citrus fruits, involves comprehensive analysis and the identification of various compounds involved in the complex biochemical pathways of a fruit [5]. These compounds play essential roles in the biochemical processes of citrus fruits and contribute to their nutritional value, taste characteristic, and potential health benefits [6]. Important metabolites of citrus fruits include sugars and sugar alcohols, which contribute to the perceived sweetness, whereas organic acids, such as citric acid and malic acid, contribute to their tartness [7]. In contrast, volatile organic compounds (VOCs) significantly affect the sensory experience of the consumer by providing citrusy aromas and enhancing the overall flavor quality of the fruits [7,8]. The complex combination of VOCs, which are composed of terpenes, alcohols, aldehydes, ketones, ethers, esters, and oxides, imparts a unique aroma profile to each citrus fruit, including *C. depressa* [9]. Our previous studies highlighted the importance of VOCs in *C. depressa* of Okinawa origin on its aroma profile, which is much different from other similar small-type citrus cultivars [1,10]. Taste-related metabolites and aroma-emitting VOCs work synergistically to create balanced sensory traits in citrus fruits [3,7].

Similar to different varieties of the same species, plant cultivation lines, i.e., distinct lineages or groups of plant species that have been selected and bred for specific desirable traits, exhibit a significant impact on fruit quality, influencing various aspects, such as appearance, flavor, nutritional composition, and disease resistance [1,6,9,11]. Each cultivation line of a fruit species is given a distinct name. For instance, the Okinawa region has the following four major *C. depressa* cultivation lines: Katsuyama kugani, Ogimi kugani, Izumi kugani, and Kaachi; each line contains different proportions of both taste- and aroma-contributing compounds [1,3]. Geographical location also exerts a profound effect on the production of metabolites and VOCs in citrus fruits and therefore their taste and aroma characteristics, owing to variations in soil composition, water quality, and microclimate [12,13]. The interplay between these geographical factors results in the diverse and distinctive flavor profiles observed in citrus fruits from different geographical cultivation areas, offering consumers a variety of flavors to savor [12,14]. Therefore, it is important to characterize these flavor-responsible substances in *C. depressa* from different origins in which the compounds may impact the flavor quality of the fruits and their derived food and beverage products.

There is currently an upsurge of interest in profiling of the metabolites and VOCs of horticultural products, including citrus fruits [11,12,13]. However, studies on the metabolites and VOCs of *C. depressa* cultivation lines from different geographical regions (Kagoshima to Taiwan) are limited. To date, the compositional analysis of *C. depressa* has been limited to the same cultivation area of those regions [1,3]. We hypothesized that different cultivation regions would affect the production of metabolites and VOCs of *C. depressa* fruits. Therefore, in the present study, we investigated the metabolite and VOC profiles of 18 *C. depressa* cultivation lines from Kagoshima, Okinawa, and Taiwan. The metabolites were analyzed using gas chromatography–time-of-flight mass spectrometry (GC-TOF-MS), and the VOCs were isolated using solid-phase microextraction (SPME) and analyzed using GC-MS. Additionally, the physicochemical properties of the fruits, such as total soluble solid (TSS) content, titratable acidity (TA), and pH, were compared. To our knowledge, this is the first report to characterize the metabolite and VOC profiles of *C. depressa* fruits from different geographical regions using analytical instrumentations and multivariate analyses, such as principal component analysis (PCA), orthogonal partial least squares-discriminant analysis (OPLS-DA), and hierarchical clustering analysis (HCA).

## 2. Materials and Methods

### 2.1. Sample Preparation

**Figure 1:**
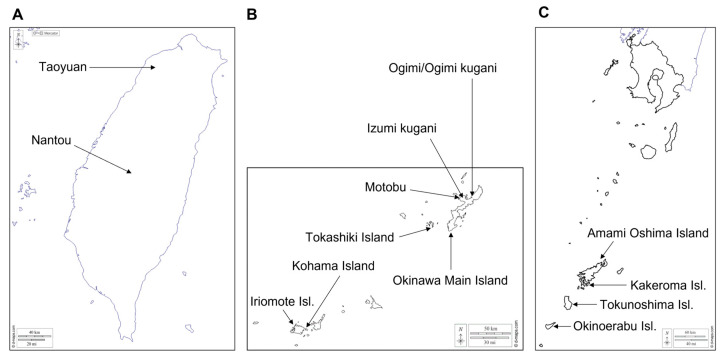
Maps of initial collection sites of *Citrus depressa* cultivation lines: (**A**) Taiwan; (**B**) Okinawa; and (**C**) Kagoshima. The maps are arranged from left to right according to their longitudinal locations (the maps were retrieved and modified from https://d-maps.com/carte.php?num_car=643, https://d-maps.com/carte.php?num_car=12237, and https://d-maps.com/carte.php?num_car=11493, respectively, assessed on 26 June 2023).

For this study, we collected mature ripe fruits of five *C. depressa* cultivation lines indigenous to Kagoshima (JP-46, geographic code for Kagoshima as defined by ISO 3166; i.e., Amami Oshima, Kakeroma, Shikunin spicy (Tokunoshima), Shikunin sweet (Tokunoshima), and Shikuribu (Okinoerabu)) and eleven of the Okinawan lines (JP-47, geographic code for Okinawa; i.e., Hijakunibu, Iriomote, Ishikunibu, Izumi kugani, Kaachi, Kohama, Motobu, Nakamoto seedless, Ogimi, Ogimi kugani, and Tokashiki) from experimental farms at the Faculty of Agriculture, Kagoshima University and the Okinawa Prefectural Agricultural Research Center Nago Branch, respectively, in December 2020. The following Okinawan lines originated from unspecified sites on the Okinawa main island: JP-47-Hijakunibu, JP-47-Ishikunibu, JP-47-Kaachi, and JP-47-Nakamoto seedless. Fruits from two Taiwanese *C. depressa* cultivation lines (TW, geographic code for Taiwan) were harvested at the mature ripened stage from the Nantou and Taoyuan regions of Taiwan in November 2021. The initial collection sites of each accession of Japanese lines and the sources of Taiwanese cultivation lines are shown in Figure 1. The climatic conditions for the cultivation of *C. depressa* in Kagoshima, Okinawa, and Taiwan, including annual average rainfall and temperature, are presented in Supplementary Table S1. Fruits from two or three biological replicates were mixed and cut (without peeling process), and the cut fruits were hand-squeezed for the analyses of physicochemical properties and VOCs. The TSS content and TA of the obtained fruit juice samples were analyzed using a PAL-BX/ACID F5 portable refractometer (Atago, Tokyo, Japan), and pH was measured using a LAQUAact D-71 pH meter (Horiba, Kyoto, Japan). The fruit juice samples were stored in sealed vials at −30 °C until VOC analysis. Fruit flesh samples from two or three biological replicates were cryo-pulverized using a multi-bead shocker (Yasui Kikai, Osaka, Japan) and lyophilized for 48 h using an EYELA FDU-2000 freeze dryer (Tokyo Rikakikai, Tokyo, Japan). The freeze-dried tissue powder was stored in sealed vials at −30 °C until metabolite profiling analysis.

### 2.2. Standards and Reagents

Stable isotope-labeled standards [^13^C_12_]-sucrose, [^13^C_5_]-proline, [^2^H_4_]-succinic acid, [^2^H_6_]-2-hydroxybenzoic acid, and [^13^C_3_]-myristic acid were purchased from Cambridge Isotope Laboratories (Tewksbury, MA, USA). Standards [_13_C_6_]-glucose and [^13^C_5_, ^15^N]-pyroglutamate were obtained from Spectra Stable Isotopes (Columbia, MD, USA). Standards [^2^H_4_]-1,4-diaminobutane and [^13^C]-4-hexadecanoic acid were obtained from C/D/N Isotopes (Pointe-Claire, QC, Canada) and Icon Isotopes (Dexter, MI, USA), respectively. Methoxylamine hydrochloride, dehydrated pyridine, ethylenediaminetetraacetic acid (EDTA), and *n*-alkanes were purchased from Sigma-Aldrich (St. Louis, MO, USA). N-Methyl-N-trimethylsilyl-trifluoroacetamide (MSTFA) and 1-hexanol were obtained from Tokyo Chemical Industry (Tokyo, Japan). All other reagents were of analytical grade.

### 2.3. Metabolite Profiling Analysis

Metabolites from the freeze-dried fruit tissue powder of *C. depressa* were extracted using an organic solvent containing stable isotope reference compounds and zirconia beads and analyzed using GC-TOF-MS [15]. Briefly, 5 mg of the lyophilized samples was added to 2 mL microtubes, which was followed by the addition of 1 mL of organic solvent (chloroform, methanol, and water; 3:1:1; *v*/*v*/*v*). The mixture was shaken at 15 Hz for 10 min using a mixer mill (Tissuelyzer; Qiagen, Venlo, The Netherlands), centrifuged at 13,000× *g* for 10 min at 4 °C, and the resulting supernatant (100 μL) was transferred to a glass-insert vial and evaporated to dryness using a Savant SPD2010 SpeedVac (Thermo Fisher Scientific, Waltham, MA, USA). The dried extracts containing metabolites were derivatized using 30 μL of methoxylamine hydrochloride (20 mg/mL in pyridine) for 23 h, which was followed by the addition of 30 μL of MSTFA; thereafter, the mixtures were incubated for 1 h at 37 °C under shaking. Subsequently, 30 μL of *n*-heptane was added to each sample. The derivatized metabolite analytes (1 μL) were injected into an Agilent 6890N GC system (Agilent Technologies, Santa Clara, CA, USA) coupled with a Pegasus 4D TOF mass spectrometer (LECO, St. Joseph, MI, USA). Chromatography was performed using a Rxi-5 Sil MS column (30 m × 0.25 mm; 0.25 μm; Restek, Bellefonte, PA, USA). The column oven temperature was set to 80 °C for 2 min, increased to 320 °C at a rate of 30 °C/min, and held for 4.5 min. Helium was used as the carrier gas at a constant flow rate of 1 mL/min. The MS ionization energy was set to 70 eV, and the MS acquisition range and rate were *m*/*z* 60–800 and 30 spectra/s, respectively. The ion source and transfer line temperatures were 200 °C and 250 °C, respectively. Retention indices (RIs) were calculated using an *n*-alkane mixture (C_8_–C_40_). The acquired non-processed MS data were converted to the NetCDF format using Chroma TOF Version 4.50 (LECO). Data pretreatment, including smoothing, alignment, and multicurve resolution (H-MCR), was performed using MATLAB Version 7.0 (MathWorks, MA, USA). Data matrices containing chromatographic peaks were normalized to that of the internal standard [^13^C]-4-hexadecanoic acid, and the metabolites were annotated using an in-house metabolite library in the Platform for RIKEN Metabolomics (PRIMe) and the reference libraries Golm Metabolome Database, NIST/EPA/NIH library (NIST 14), and Adam’s Library (3rd and 4th editions). All analyses were performed in triplicate.

### 2.4. VOC Analysis

The VOCs in the juice squeezed from *C. depressa* fruits were analyzed using SPME-GC-MS [16]. Briefly, juice (2 mL), internal standard 1-hexanol (10 µL, 0.05%, *w*/*v*), and EDTA (1 mL, 150 mM, pH 7.5) were added to a sealed 20 mL vial, and the mixture was heated at 40 °C for 5 min. VOCs were extracted using a divinylbenzene/carboxen/polydimethylsiloxane 50/30 μm SPME fiber (Supelco, Bellefonte, PA, USA) at 40 °C for 10 min under continuous agitation using a CombiPAL autosampler (CTC Analytics, Zwingen, Switzerland). The SPME fiber was injected into a 7890 B GC-5977A MS system (Agilent Technologies) at a split ratio of 10:1 for 2 min. The inlet temperature was set at 250 °C, and helium was used as the carrier gas at a constant flow rate of 1 mL/min. A DB 5MS column (30 m × 0.25 mm, 0.25 μm; Agilent Technologies) was used for analyses, and the column oven was programmed to start from 55 °C and reach 200 °C at a rate of 3 °C/min (without any hold time). MS acquisition was performed for *m*/*z* 33–450. MS ionization was performed in electron ionization (EI) mode (70 eV). The ion source and transfer line temperatures were both set to 230 °C. Non-processed MS data were converted to the NetCDF format, and the chromatographic peaks were aligned and annotated using MS-DIAL Version 4.8 (RIKEN, Kanagawa, Japan). VOCs were identified based on linear Ris upon measurement of the homologous series of *n*-alkanes (C_7_–C_30_), which was followed by comparison of the MS patterns of the peaks with the MS data obtained from the GC-MS DB-Public-KovatsRI-VS3 and the NIST/EPA/NIH library (NIST 14). All analyses were performed in triplicate.

### 2.5. Statistical Analysis

Data matrices of metabolites and VOCs were normalized using log_10_-transformation and unit variance scaling, and multivariate analyses, namely PCA, OPLS-DA, and HCA, were performed using SIMCA Version 17 (Sartorius, Göttingen, Germany). Significant metabolites were selected based on log_2_-fold change (FC) and false discovery rate (FDR) indicators, which were processed using R Version 3.6.2 in the “LIMMA” package (https://bioconductor.org/packages/release/bioc/html/limma.html, assessed on 18 November 2022) [15,17]. The volcano visualization plot was outlined by comparing the FC values of the metabolites between two groups (cultivation regions) on the *x*-axis and their statistically significant differences on the *y*-axis.

## 3. Results

### 3.1. Physicochemical Properties of C. depressa Fruits

**Figure 2:**
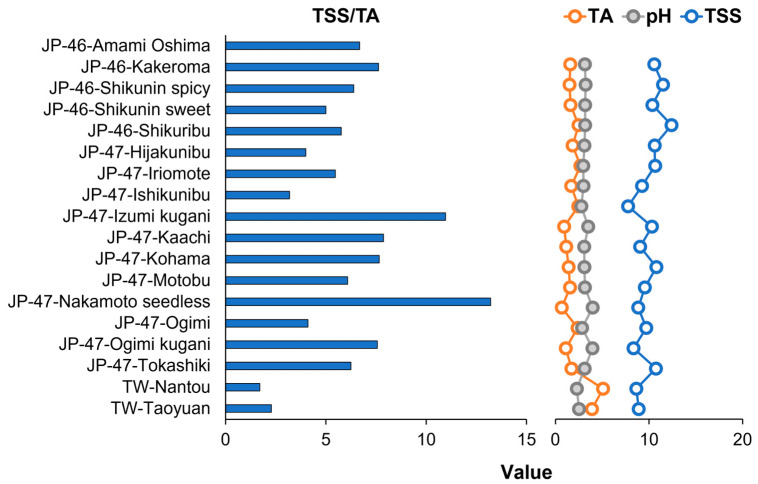
Physicochemical properties (TSSs: total soluble solids; TA: titratable acidity; TSS/TA, and pH) of *Citrus depressa* fruits from different cultivation lines and geographical regions.

The TSS content in the extracts of mature ripe fruits of 18 C. *depressa* cultivation lines from different geographical regions was 7.75–12.4 °Brix, and JP-46-Shikunin sweet and JP-47-Ishikunibu had the highest and the lowest TSS content, respectively (Figure 2). The TW lines (TW-Nantou and TW-Taoyuan) had the highest TA values of 5.08 and 3.91%, respectively, whereas the lowest TA values were observed in the Okinawan lines: JP-47-Nakamoto seedless (0.67%), JP-47-Izumi kugani (0.94%), and JP-47-Ogimi kugani (1.11%). Moreover, the highest and the lowest pH values were observed for JP-47-Nakamoto seedless (3.98) and TW-Nantou (2.30) lines, respectively. Hence, the average TSS/TA ratios were 6.94, 6.29, and 1.99 in Okinawa-, Kagoshima-, and Taiwan-grown *C. depressa*, respectively. The TSS/TA ratio of the JP-47-Nakamoto seedless line was more than that of the other cultivation lines (13.21), whereas TW-Nantou exhibited the lowest ratio (1.70).

### 3.2. Metabolite Profiles of C. depressa Fruits

**Figure 3:**
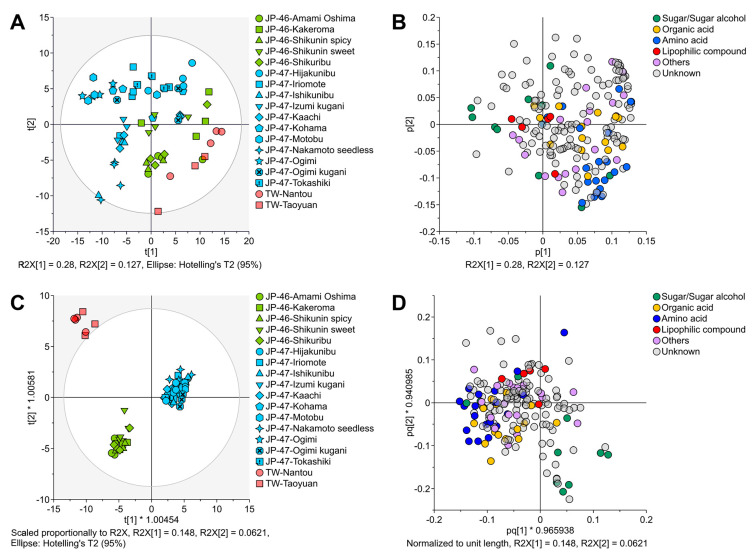
Multivariate analysis plots of metabolites of *Citrus depressa* fruits: (**A**) Principal component analysis (PCA) scores; (**B**) PCA factor loadings; (**C**) Orthogonal partial least squares-discriminant analysis (OPLS-DA) scores; and (**D**) OPLS-DA factor loadings.

Untargeted GC-TOF-MS analysis of the extracts of *C. depressa* fruits from different cultivation lines and regions revealed 192 chromatographic peaks, of which 68 metabolites, including sugars, sugar alcohols, organic acids, amino acids, and lipophilic compounds, were annotated (Supplementary Table S2). These metabolites were annotated and quantified based on their specific ion masses in which the mass intensity of a compound does not always align with its total ion chromatogram (summed signal intensity), and therefore, a numerical comparison between compounds could not be explicitly measured. Instead, the relative concentration data showed predominant compounds on the chromatograms including sugars (sucrose, glucose, and fructose) and organic acids (citric acid and malic acid). To elucidate the differences between the cultivation lines and areas, all chromatographic peaks were analyzed using multivariate analysis with unsupervised PCA and supervised OPLS-DA (Figure 3). The PCA scatter plots of the metabolites exhibited the first and second principal components (PCs) at 28.0 and 12.7%, respectively (Figure 3A,B). The PCA score plot revealed rough separation among the three cultivation regions, in which the *C. depressa* cultivation lines from Kagoshima were outlined between the Okinawan and the TW lines (Figure 3A). The PCA loading plot indicated that the amounts of sugars, sugar alcohols, and lipophilic compounds influenced the differentiation between the cultivation lines from Okinawa and Kagoshima, whereas amino acid levels tended to be responsible for the separation of the TW lines from those of other regions (Figure 3B).

OPLS-DA further differentiated the metabolites of *C. depressa* fruits and exhibited an assemblage of the three cultivation regions as objective variables for the first two predictive components (Figure 3C). Moreover, the TW lines deviated outside the 95% confidence interval of the predictive score plots, suggesting significant differences in their metabolite compositions compared to those of *C. depressa* fruits from Kagoshima and Okinawa. The OPLS-DA factor loadings were scattered, similar to the PCA factor loadings for plots of certain metabolites, and their directions were similar to those of the cultivation lines and regions (Figure 3A,B vs. Figure 3C,D). The results indicated that *C. depressa* fruits from Kagoshima and Okinawa had higher amounts of sugars/sugar alcohols, whereas the TW lines contained higher amounts of amino acids. Furthermore, OPLS-DA was used to estimate the variable importance in projection (VIP) value of each plotted chromatographic peak, including the peaks of the annotated metabolites. Variables with VIP values >1 were considered influential in the OPLS-DA model; thus, they were categorized as discriminant metabolites.

**Figure 4:**
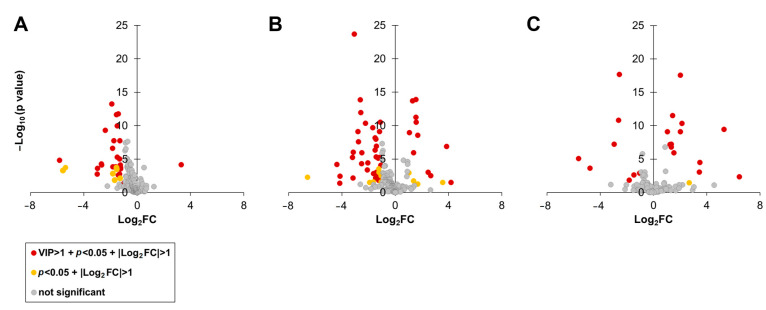
Volcano plots of metabolites extracted from *Citrus depressa* fruits: (**A**) Comparison between fruits from Okinawa and Kagoshima regions; (**B**) Comparison between fruits from Okinawa and Taiwan regions; and (**C**) Comparison between fruits from Kagoshima and Taiwan regions.

Volcano plot visualizations revealed that the metabolites contributed to the differentiation of *C. depressa* based on its cultivation area (Figure 4). The metabolites of *C. depressa* fruits from two regions were considered significantly different based on the following three parameters: *p* value of FDR < 0.05, |log_2_FC| > 1, and VIP value > 1. Leucine levels in the Okinawan *C. depressa* lines were significantly higher than those observed in Kagoshima lines (log_2_FC = 3.28), whereas the Okinawan lines had significantly lower contents of the following 12 metabolites: isoleucine, 3-amino-piperidin-2-one, methionine, tyrosine, ribose, shikimate, serine, alanine, valine, 2-oxo-glutaric acid, caffeic acid, and homoserine (Figure 4A; Supplementary Table S3). Moreover, the Okinawan lines had significantly higher amounts of sugars and sugar alcohols, including galactinol, trehalose, glucose, xylose, fructose, and sucrose (log_2_FC = 2.65, 1.68, 1.57, 1.37, 1.29, and 1.07, respectively) than the TW cultivation lines, but lower amounts of ribose and amino acids, such as isoleucine, serine, and alanine (Figure 4B; Supplementary Table S3). In contrast, the Kagoshima cultivation lines had significantly higher levels of galactinol, trehalose, xylose, sucrose, glucose, and fructose (log_2_FC = 3.44, 2.13, 2.01, 1.43, 1.33, and 1.04, respectively), whereas the TW lines had significantly higher levels of leucine and serine (Figure 4C; Supplementary Table S3).

### 3.3. VOC Profiles of C. depressa Fruits

**Figure 5:**
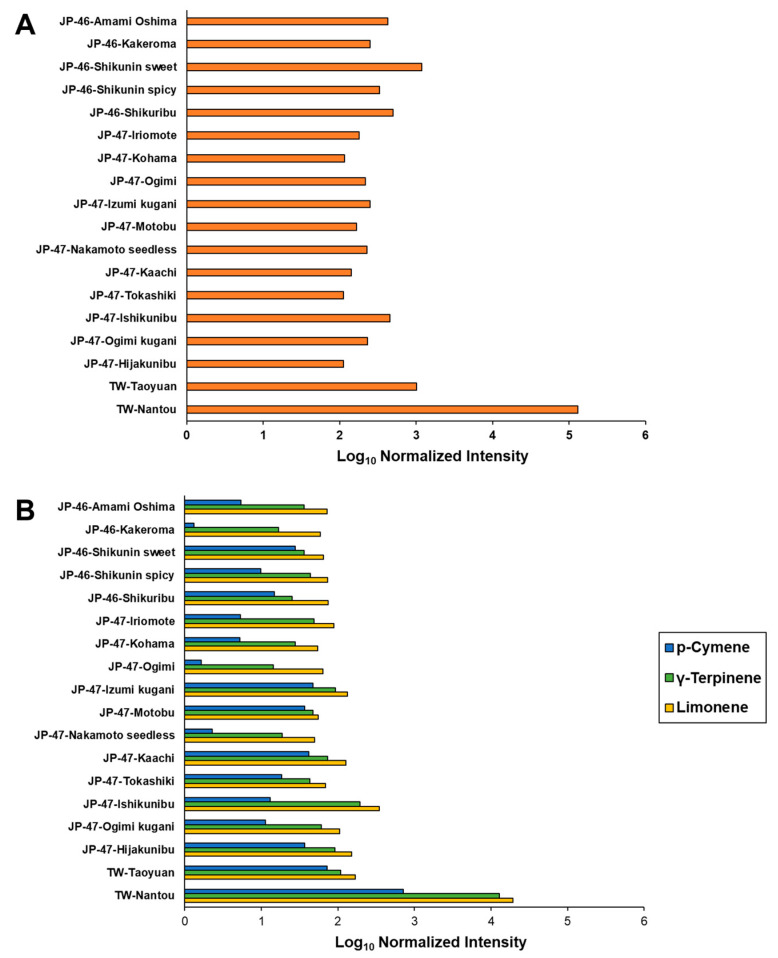
Log_10_ normalized intensities of volatile organic compounds (VOCs) present in *Citrus depressa* fruits: (**A**) All detected chromatographic peaks; and (**B**) Limonene, *γ*-terpinene, and *p*-cymene.

Untargeted GC-MS analysis of the extracts of *C. depressa* fruits belonging to different cultivation lines and regions revealed 281 chromatographic peaks corresponding to VOCs, of which 89 were annotated (Supplementary Table S4). Among all cultivation lines, the highest total log_10_ normalized concentration of the VOCs was observed in the TW-Nantou line (5.11), which was followed by JP-46-Shikunin sweet and TW-Taoyuan lines (3.08 and 3.01, respectively) (Figure 5A). Log_10_ normalized intensities of the VOCs of the other lines were 2.05–2.70 with the lowest value exhibited by JP-47-Hijakunibu. Although variations were observed in the composition of the major compounds derived from the monoterpene hydrocarbon class, the predominant VOC in these lines was limonene, which was followed by *γ*-terpinene and *p*-cymene (Figure 5B). Owing to the higher intensities of VOCs in the TW-Nantou line, it was excluded from further multivariate analyses.

**Figure 6:**
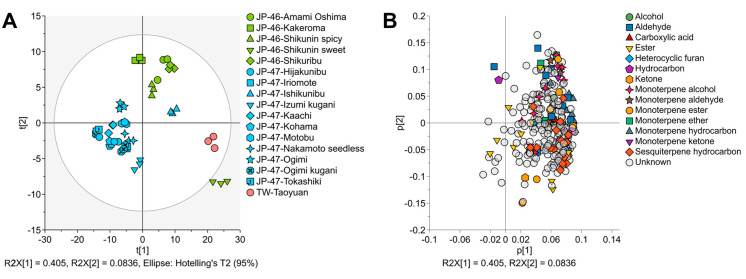
Principal component analysis (PCA) plots of volatile organic compounds (VOCs) extracted from *Citrus depressa* fruits: (**A**) PC scores; and (**B**) Factor loadings.

The PCA score plots of VOCs revealed a rough separation pattern between most of the *C. depressa* cultivation lines from Kagoshima and Okinawa in the first two PCs (PC1, 40.5%; PC2, 8.4%) (Figure 6A). The factor loadings displayed the accumulation of almost all chromatographic peaks, including those of VOCs and unknowns, as factor loadings on the positive side of PC1, as shown in the score plots of JP-46-Shikunin sweet and TW-Taoyuan (Figure 6A,B). The results indicated distinct separations of the two cultivation lines because they possessed higher total normalized chromatographic intensities than the other plotted cultivation lines as per PCA (Figure 5 vs. Figure 6). The PC1 axis of factor loadings indicated that these lines tended to draw plots of VOCs in the positive direction of the principal component because they contained higher intensities of VOCs, especially monoterpene hydrocarbons and sesquiterpene alcohols. Conversely, the normalized intensities of the other cultivation lines were lower, which was responsible for the clear separation in the negative direction of PC1. Moreover, the factor loadings of monoterpene alcohols, including linalool, α-terpineol, citronellol, and thymol, may be responsible for the differentiation of *C. depressa* cultivation lines from Kagoshima, whereas esters, such as isopentyl acetate, methyl hexanoate, and methyl heptanoate, may be related to Okinawan *C. depressa*.

**Figure 7:**
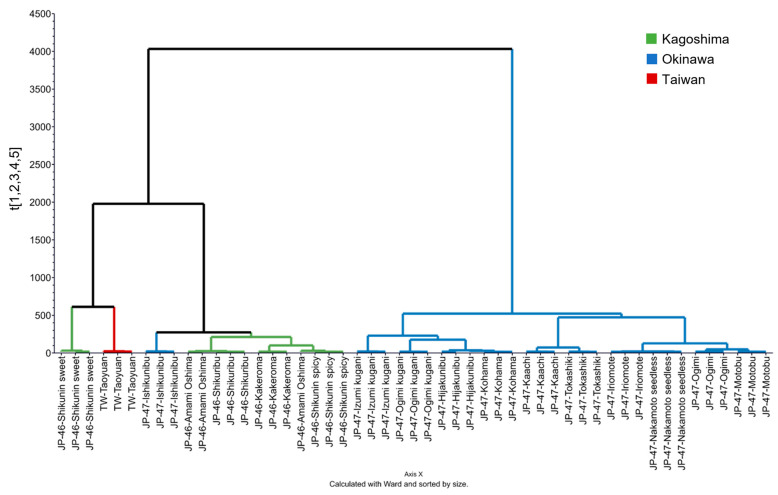
Hierarchal clustering analysis (HCA) dendrogram according to volatile organic compounds (VOCs) present in *Citrus depressa* fruits.

The HCA dendrogram further elucidated the differentiation of *C. depressa* based on VOCs (Figure 7). The dendrogram plot shows the following three main groups (from left to right): (1) JP-46-Shikunin sweet and TW-Taoyuan groups, (2) Okinawan Ishikunibu and other Kagoshima-grown line groups, and (3) other Okinawan line groups. The last group, which consisted of ten cultivation lines, was micro-clustered into several branches, such as the JP-47-Hijakunibu and JP-47-Kohama subgroups, JP-47-Kaachi and JP-47-Tokashiki subgroups, and JP-47-Iriomote and JP-47-Nakamoto seedless subgroups. Thus, HCA provided separation in more detail than PCA, particularly for the cultivation lines that were plotted at overcrowded locations for both negative factors of PC1 and PC2 (Figure 6 vs. Figure 7).

## 4. Discussion

The cultivation lines and geographical areas had notable effects on the quality of *C. depressa* fruits (Figure 2). The basic physicochemical properties, such as TSS content, TA, and pH, can determine the characteristics of a fruit. The TSS/TA ratios of *C. depressa* fruits from Okinawa and Kagoshima regions, on average, were comparable to the TSS/TA ratio of grapefruit (6.92), but they were lower than those of mandarin (8.00–16.87), sweet orange (8.23–14.63), and pummelo (9.57–17.09) [6,18,19]. However, some exceptions were observed, such as the TSS/TA ratios of JP-47-Nakamoto seedless and JP-47-Izumi kugani lines, which were higher (13.21 and 10.96, respectively). In contrast, the characteristics of the TW lines were similar to those of lemon (1.79–2.20) and bitter orange (1.93) [18,19]. These physicochemical properties, particularly the TSS/TA ratio, which represents the balance between soluble solid content and acidity of the fruits, can provide information regarding harvest timing, post-harvest handling, storage conditions, and processing methods to many stakeholders, including cultivators, processors, and consumers [20,21]. The Japanese and TW *C. depressa* fruits in the current study were collected at the mature ripe stage; during this stage, the local harvesters commonly collect the fruits and commercially distribute them as ripe fruits. However, their botanical ripening development might slightly fluctuate among the cultivation lines, thus affecting their physicochemical traits. Nonetheless, the results provide basic physicochemical characterization of the aggregated soluble solids and acidity-related compounds of the fruits; these traits have been considered as the overall quality parameters for primary classification by farmers, but the results do not elucidate the composition and key compounds responsible for the flavor quality of citrus fruits, including *C. depressa* fruits [18,21]. Therefore, a quantitative determination of taste-related compounds, either traditional or advanced analytical technology, such as chromatography techniques, is required.

Advanced analytical instrumentation techniques for metabolite and VOC profiling are required to elucidate the factors that influence the flavor quality of *C. depressa* fruits from different cultivation lines and regions (Supplementary Tables S2 and S4). The metabolites detected in *C. depressa* fruits were consistent with those previously reported [5,12]. Metabolites such as sugars and sugar alcohols (glucose, galactose, and myo-inositol), organic acids (isocitrate, succinate, and 2-oxoglutaric acid), amino acids (alanine, valine, glycine, serine, threonine, and proline), and lipophilic compounds (octadecanoic acid and hexadecanoic acid) are also reported in other citrus species, including pummelo, navel orange, ponkan, grapefruit, Satsuma mandarin, and lemon [22,23,24,25,26]. The metabolite composition of citrus fruits differs depending on their genetic variability, including differences in varieties and cultivation lines [5,18]. Feng et al. reported that metabolite profiles could distinguish some varieties of mandarin oranges and that the orchard location affected the overall metabolite profile of fruits of the same variety [12].

Multivariate analyses revealed variations in the primary and other accumulated metabolites extracted from the *C. depressa* fruits of the three cultivation regions (Figure 3). The VIP value of the OPLS-DA calculation elucidated the importance of the metabolites (variables) in differentiating cultivation areas, with higher VIP values suggesting higher importance (Supplementary Table S3). Volcano plots provided a visual representation of the statistical significance and FCs in metabolite concentrations between two cultivation regions (Figure 4). In these plots, the threshold (>1) of the VIP value was set to reveal the metabolites that significantly contributed to the observed differences, which were identified as potential chemical markers. These results indicate that *C. depressa* fruits from Kagoshima and Okinawa had higher sugar and sugar alcohol contents than those of the TW lines, and these compounds might be responsible for imparting a sweet taste to the fruits [27]. In contrast, the TW *C. depressa* fruits had higher leucine, isoleucine, serine, and alanine levels. At certain concentrations, these amino acids provide taste perception to humans, such as alanine and leucine impart sweetness and bitterness, respectively; however, the taste imparted by isoleucine and serine is barely perceptible [28]. Moreover, their concentrations and thresholds are not comparable to those of other citrus taste-active compounds that can provide stronger perceivable sensory sensations, such as sugars (sweetness) and secondary metabolites, such as flavonoids and terpenoids (bitterness) [29].

The variation in the concentrations of these key metabolites of *C. depressa* fruits can be attributed to a combination of genetic factors (cultivation line), including metabolic regulation and environmental conditions (cultivation area) [5,12,25]. Genetic variation plays a crucial role because different cultivation lines possess unique genetic traits that influence the activity of enzymes involved in carbohydrate and amino acid metabolism [6,20]. The regulation of metabolic pathways, including the expression of key genes and enzyme activities, differs between cultivars, leading to variations in sugar synthesis, breakdown, transport, and amino acid biosynthesis [6]. Additionally, the environmental factors, such as temperature, light, and nutrient availability, of the three cultivation regions (Kagoshima, Okinawa, and Taiwan) can affect metabolic processes and influence the abundance of sugars and amino acids [11,25]. Therefore, future studies on the interplay between these factors can elucidate the reason behind variations in the sugar, sugar alcohol, and amino acid levels of different *C. depressa* cultivation lines, thereby providing valuable insights for breeding programs and fruit quality improvement.

In the present study, some cultivation lines possessed higher concentrations of total VOCs, such as the TW-Nantou and -Taoyuan lines and the Shikunin sweet line from Kagoshima, than those from Okinawa (Figure 5 and Figure 6). Lin et al. reported that the expression of terpene synthase genes, particularly limonene and α-terpineol synthases, is weaker in the Okinawan cultivation lines than in the TW cultivation lines [2]. However, the key compounds of these cultivation lines were monoterpene hydrocarbons, such as limonene, *γ*-terpinene, and *p*-cymene. This is in accordance with the results of previous studies on the VOC composition of the essential oils of Okinawan *C. depressa*, and they may potently emit the following odors with high flavor dilution factors: citrus and minty (limonene); chewing gum, oily, and waxy (*γ*-terpinene); and green, woody, and herbaceous (*p*-cymene) [10,30]. However, multivariate analysis via the PCA plot also indicated higher intensities of monoterpene alcohols, such as linalool, α-terpineol, and citronellol, in the *C. depressa* from Kagoshima (Figure 6). Linalool is one of the most aroma-active compounds in citrus species, including Okinawan *C. depressa*, and it imparts citrus, floral, and sweet aromas [10,31]. Other monoterpene alcohols also provide important odors to fruits, such as green, floral, and sweet (α-terpineol), and citrus, fresh, and sweet (citronellol) [10]. Furthermore, the aroma traits of Okinawan cultivation lines may be supplemented with higher amounts of ester components such as isopentyl acetate, methyl hexanoate, and methyl heptanoate, and they may provide various fruity aromas to the fruits [32]. Additionally, HCA provided a clustering plot for identifying similar patterns of *C. depressa* with different origins based on the VOC dataset (Figure 7). Along with the PCA plot, the dendrogram also provided insights into the similarities and dissimilarities between cultivation lines, aiding in the classification and interpretation of VOC profiling data [30].

VOC profile data are crucial for predicting and assessing the flavor quality of *C. depressa* fruits and their derivatives. These outcomes provide insights into the possible aroma profile, flavor development, quality assessment, and consumer preferences [33]. By leveraging this information, agro-industrial stakeholders in the region can make informed decisions throughout the *C. depressa* supply chain, ultimately enhancing the overall sensory experiences and satisfaction of the consumers [33,34]. For instance, in the development of new products and the optimization of existing products, food and beverage manufacturers can tailor formulations to enhance the specific aroma profiles of *C. depressa* products and meet consumer preferences in these regions. VOC data can also guide the selection of *C. depressa* cultivation lines for targeted applications, ensuring that the final products exhibit the desired flavor characteristics [33]. Therefore, the results of the present study provide important data for further sensory evaluation studies of *C. depressa* cultivation lines in different regions.

The metabolite and VOC data can be used to assess the post-harvest quality of *C. depressa* fruits, especially for providing information regarding their taste- and aroma-responsible substances, respectively. However, the mechanism underlying genetic regulation and metabolic pathways for determining the production of VOCs in citrus fruits, and thus the association between metabolites and VOCs, are still largely unknown [2,16]. These data, nevertheless, will help in the optimization of postharvest handling practices, storage conditions, and transportation methods to maintain fruit quality and extend shelf life. The cumulative effect of specific cultivation lines also contributed to the distinctive metabolite and VOC profiles observed in *C. depressa* fruits from Kagoshima, Okinawa, and Taiwan. Previous studies have suggested the contributions of the above-mentioned metabolites and VOCs on taste and aroma characteristics of several Okinawan lines [3,10]. Therefore, understanding the effect of cultivation area on *C. depressa* flavor is essential for both cultivators and consumers. This enables the cultivators to select suitable cultivation lines for specific regions and optimize growing conditions to enhance flavor development. Similarly, consumers can appreciate and explore the diverse flavor qualities offered by *C. depressa* fruits from different cultivation areas, enriching their culinary experiences and preferences.

## 5. Conclusions

The fruits of 18 *C. depressa* cultivation lines from Kagoshima, Okinawa, and Taiwan varied in physicochemical properties, metabolites, and VOCs. Multivariate plots developed via PCA and OPLS-DA elucidated on the differentiation of metabolite profiles of *C. depressa* lines based on their cultivation region. Kagoshima- and Okinawa-grown *C. depressa* contained higher contents of sugars and sugar alcohols such as galactinol, trehalose, xylose, glucose, sucrose, and fructose (log_2_FC = 2.65–3.44, 1.68–2.13, 1.37–2.01, 1.33–1.57, 1.07–1.43, and 1.04–1.29, respectively), whereas the TW lines contained a higher content of amino acids, including leucine, isoleucine, serine, and alanine. The untargeted SPME-GC-MS analysis and multivariate analysis also revealed variations in the VOC profiles of the *C. depressa* cultivation lines, wherein remarkably high intensities of VOCs were recorded in some lines, such as Nantou (Taiwan), Shikunin sweet (Kagoshima), and Taoyuan (Taiwan) (log_10_ normalized concentration = 5.11, 3.08, and 3.01, respectively). Moreover, unlike other Okinawan cultivation lines, Ishikunibu had a VOC profile comparable to that of Kagoshima lines. These results suggest that the formation of metabolites and VOCs in *C. depressa* fruit is highly affected by both cultivation line and origin. This study also elucidates potential chemical markers of taste and palatability in *C. depressa* from different cultivation origins. Both cultivation lines and regions influence the aroma-emitting VOCs and flavor-associated metabolites of *C. depressa* and determine the potential flavor quality. Thus, the relationship between metabolites and VOCs and the sensory characteristics of *C. depressa* fruits from different regions should be investigated in future studies to determine which cultivation lines can be used as raw materials for the production of food and beverage products, including their taste and aroma traits. Moreover, the characterization of more preference- and biofunctionality-related metabolites, particularly secondary metabolites such as carotenoids and flavonoids, of *C. depressa* from Kagoshima, Okinawa, and Taiwan should be further investigated.

## Data Availability

Data are contained within the article and Supplementary Materials.

## Supplementary Materials

The following supporting information can be downloaded at: https://www.mdpi.com/article/10.3390/foods12152951/s1: Table S1. Climatic conditions in the cultivation area of *Citrus depressa*; Table S2. Metabolites of *Citrus depressa* from different geographical origins; Table S3. Comparison of log2-fold changes, false discovery rates, and variable importance in the projection values of significant metabolites of *Citrus depressa* fruits; Table S4. Volatile organic compounds in *Citrus depressa* from different geographical origins.
